# Im Schatten der Paraden: Erinnerungen an eine instrumentalisierte Psychiatrie in der DDR

**DOI:** 10.1007/s00115-025-01892-w

**Published:** 2025-08-28

**Authors:** Ekkehardt Kumbier, Lillian Osel, Olaf Reis, Kathleen Haack

**Affiliations:** 1 Arbeitsbereich Geschichte der Medizin, Universitätsmedizin Rostock, Doberaner Straße 140, 18057 Rostock, Deutschland; 2 Klinik für Psychiatrie, Neurologie, Psychosomatik und Psychotherapie im Kindes- und Jugendalter, Universitätsmedizin Rostock, Rostock, Deutschland

**Keywords:** Medizingeschichte, Ostdeutschland, Einweisung, Zeitzeugen, Qualitative Inhaltsanalyse, History of medicine, East Germany, Commitment, Contemporary witnesses, Qualitative content analysis

## Abstract

**Zielstellung:**

Anhand von Zeitzeugenaussagen wird untersucht, wie Mitarbeitende in den psychiatrischen Einrichtungen der Deutschen Demokratischen Republik (DDR) mit psychisch kranken Menschen zu gesellschaftlichen Ereignissen umgingen.

**Methodik:**

Es wurden 63 Zeitzeugen befragt, die in psychiatrischen Einrichtungen in der DDR beruflich tätig waren. Die Interviews wurden qualitativ ausgewertet.

**Ergebnisse:**

Die Interviews zeigen, dass es medizinisch nicht indizierte Beschränkungen und Einweisungen zu bestimmten gesellschaftlichen Ereignissen wie Staatsfeiertagen oder Staatsbesuchen gab. Mit den Anweisungen wurde unterschiedlich umgegangen. Bei Nichtbeachtung werden überwiegend keine schwerwiegenden Konsequenzen beschrieben.

**Schlussfolgerung:**

Menschen mit psychischen Erkrankungen sollten zu bestimmten gesellschaftlichen Ereignissen von der Öffentlichkeit ferngehalten werden. Das Personal in den psychiatrischen Einrichtungen ging unterschiedlich damit um. Das weist auf einen Handlungsspielraum hin. Die Entfernung aus der Gesellschaft stellt eine Stigmatisierung psychisch kranker Menschen dar. Die Psychiatrie in der DDR wurde dahingehend auch in politischer Hinsicht instrumentalisiert.

## Abstract (en)

**Objective:**

Based on contemporary witness statements, the study examines how employees in the psychiatric institutions of the German Democratic Republic (GDR) dealt with mentally ill people at social events.

**Methodology:**

In this study 63 contemporary witnesses who worked in psychiatric institutions in the GDR were interviewed. The interviews were evaluated qualitatively.

**Results:**

The interviews show that there were restrictions and admissions for certain social events, such as national holidays or state visits, that were not medically indicated. These instructions were handled differently. Noncompliance with these instructions was largely not reported as having serious consequences.

**Conclusion:**

People with mental illnesses were supposed to be kept away from public events during certain social events. The staff in psychiatric institutions dealt with this differently. This indicates a certain degree of flexibility. The removal from society represents a stigmatization of mentally ill people. Psychiatry in the GDR was also instrumentalized for political purposes.

## Einleitung

In der historischen Forschung zur Psychiatrie in der Deutschen Demokratischen Republik (DDR) wurde zunächst der Frage nach einem möglichen Missbrauch nachgegangen. Den Hintergrund bildeten die Erfahrungen in der Sowjetunion, wo Dissidenten für psychisch krank erklärt und gegen ihren Willen in psychiatrische Kliniken weggesperrt worden waren [13]. Ähnliches stellte man sich auch in der SED-Diktatur vor [7]. Bis heute finden sich jedoch keine Hinweise, dass die Psychiatrie systematisch zur Verfolgung politischer Gegner genutzt wurde. Nichtsdestotrotz gab es Formen des Missbrauchs [2, 16]. Wenig ist bislang über medizinisch nicht gerechtfertigte Einweisungen und Ausgangsbeschränkungen von psychisch kranken Menschen zu öffentlichen Anlässen bekannt. Vor allem Loos, Süß und Erices haben darauf hingewiesen [2, 10, 17].

Dabei übt die Psychiatrie nicht nur in sozialistischen oder als diktatorisch definierten Staaten ordnungspolitische Funktionen aus. Auch in demokratischen Gesellschaften ist ihr Auftrag ein doppelter: einerseits Ordnung und Sicherheit zu gewährleisten und andererseits die Behandlung psychisch Kranker zu garantieren. Dies kann zu Eingriffen in die Freiheitsrechte von Menschen führen. Das Ausmaß der ordnungspolitischen Aufgaben ist allerdings gesellschaftsabhängig, sowohl hinsichtlich der Entscheidungen der Machthaber als auch hinsichtlich des Umgangs professioneller Akteure damit.

Im Folgenden soll gezeigt werden, wie sich Mitarbeitende in der Psychiatrie der DDR in Situationen verhielten, in denen der Staat versuchte, Einfluss zu nehmen. Die Frage nach der Umsetzung staatlicher Vorgaben wie auch die Verknüpfung zwischen Psychiatrie und autoritärem System der DDR ist hier relevant. Konkret geht es um das Verhältnis von Individuen und Herrschaft auf der Ebene der Psychiatrie aus Sicht einzelner Akteure. Insbesondere interessierte uns, wie die in der Psychiatrie Tätigen mit psychisch kranken Menschen zu bestimmten Anlässen umgingen. Was geschah mit jenen, die anlässlich von gesellschaftlichen Ereignissen in psychiatrische Kliniken eingewiesen oder dort zurückgehalten wurden, obwohl dafür keine medizinische Notwendigkeit bestand? Wie wurden diese Handlungen begründet und woher kamen die entsprechenden Anweisungen? Drohten Konsequenzen, wenn sie missachtet wurden? Hatten die Mitarbeitenden einen Handlungsspielraum? Diese Fragen sollen anhand der Berichte von Zeitzeuginnen und Zeitzeugen untersucht werden.

## Methodik

In dem Projekt „Psychiatrie in der DDR zwischen Hilfe, Verwahrung und Missbrauch?“ des Verbundprojekts „Seelenarbeit im Sozialismus“ wurden zwischen Februar 2020 und August 2021 insgesamt 25 Ärzte und Ärztinnen, 11 Psychologen und Psychologinnen, 9 Fürsorger und Fürsorgerinnen und 18 Pflegekräfte interviewt, die in ambulanten oder stationären psychiatrischen Einrichtungen der DDR beruflich tätig waren. Die Kontaktaufnahme erfolgte über bestehende akademische und medizinische Netzwerke sowie über direkte Empfehlungen von beruflichen Partnern. Nach einer unverbindlichen Anfrage erhielten potenzielle Teilnehmer ausführliche Informationen zum Projekt und eine Einwilligungserklärung.

Die Studie war von der Ethikkommission der Universitätsmedizin Greifswald befürwortet worden (EK Votum BB 062/20). Aufgrund der COVID-19-Pandemie wurden die leitfadenbasierten Interviews telefonisch durchgeführt. Die Audiodateien wurden durch eine externe Schreibkraft anhand vereinfachter Transkriptionsregeln verschriftlicht. Die Auswertung erfolgte computergestützt mithilfe des Analyseprogramms für qualitative Daten MAXQDA [11]. Dabei wurde die inhaltlich strukturierende Inhaltsanalyse nach Kuckartz angewendet, die ein induktiv-deduktives Vorgehen erlaubt [6]. Im ersten Schritt erfolgte die Zuordnung aller Textsegmente (Codierungen) zu den Teilaspekten „Anlässe“, „Einweisung“ und „Entlassungsmanagement“. Während der Auswertung wurde deutlich, dass sich zusätzliches Material für die Bildung einer neuen Kategorie „Umgang mit Anweisungen“ fand, die zunächst den „Anlässen“ zugeordnet wurde. Unter der Codierung „Anlässe“ finden sich 67 Aussagen von 44 Befragten und unter „Einweisung“ 85 Äußerungen von 52 Personen. Dem Aspekt „Entlassungsmanagement“ konnten 135 Aussagen von 60 Interviewten zugeordnet werden. Einige Befragte äußerten sich mehrmals. In der weiteren Auswertung wurde die Kategorie „Anlässe“ zur Spezifizierung in sechs weitere Unterkategorien unterteilt, um zu erfahren, zu welchen Anlässen es Ausgangsbeschränkungen respektive anlassbezogene Einweisungen gegeben hatte. Nach nochmaliger Überprüfung der Interviews konnten die in Tab. 1 ersichtlichen Kategorien erstellt werden. Auch wurde differenziert, wie die beruflichen Akteure mit Anweisungen zu Beschränkungen des Ausgangs von Patientinnen und Patienten sowie Aufforderungen zur Aufnahme von Personen zu den Anlässen umgingen, ob es Zuwiderhandlungen gab und inwieweit diese Konsequenzen nach sich zogen. Um das darstellen zu können, wurde die Kategorie „Umgang mit Anweisungen/Konsequenzen“ etabliert. Diesem Subcode ließen sich 46 Äußerungen aus 28 Interviews zuordnen. Nach den Codierungen wurden die Aussagen mittels Summary Grid in MAXQDA paraphrasiert. Die Ergebnisse werden im Folgenden dargestellt und mit Zeitzeugenaussagen unterlegt. Die Angaben beziehen sich auf die 1970er- und 1980er-Jahre.

**Tab. 1:** Nennung von Anlässen für Ausgangsbeschränkungen oder Vorsorgeeinweisungen, getrennt nach Berufsgruppen (*n* = Anzahl der Sprecher)

Berufsgruppe	Anlass			
	Feiertag	Staatsbesuch	Wahl	Messen/Feste/Spiele
Ärzte	13 (*n* = 10)	9 (*n* = 7)	4 (*n* = 3)	5 (*n* = 3)
Psychologen	11 (*n* = 8)	5 (*n* = 5)	2 (*n* = 2)	1 (*n* = 1)
Fürsorger	5 (*n* = 5)	9 (*n* = 5)	–	–
Pflege	16 (*n* = 10)	7 (*n* = 6)	3 (*n* = 2)	1 (*n* = 1)
Zitate gesamt	45 (*n* = 33)	30 (*n* = 23)	9 (*n* = 7)	7 (*n* = 5)

## Ergebnisse^1^

### Anlässe und Begründung für Anweisungen

Die beruflichen Akteure wurden befragt, ob es Anlässe gegeben habe, zu denen Patientinnen und Patienten in die Psychiatrie eingewiesen werden sollten oder den bereits in stationärer Behandlung Befindlichen kein Ausgang gewährt wurde. Hierbei ließen sich die in Tab. 1 genannten Anlässe finden, die von den Ärzten, Psychologen, Fürsorgern und Schwestern bzw. Pflegern genannt wurden. Die Anlässe wurden nach der Gesamtzahl der codierten Zitate geordnet.

Eine Ärztin aus Arnsdorf gab an, man habe „sich eigentlich regelrecht darauf vorbereiten (können) […] vor bestimmten Feiertagen^2^ […], weil da wusste man, dass im Prinzip alle möglichen unliebsamen und auffälligen Personen eingewiesen wurden“ (056_A_21-03-05: 36). Eine Stralsunder Psychologin bestätigte, dass „vor bestimmten Feiertagen […] Betten freigehalten werden sollten für Menschen, die randalierten oder die aufbegehrten in der Stadt, und Patienten auch keinen Ausgang bekamen“ (028_Psy_20-08-27: 175). Eine Fürsorgerin aus Halle berichtete: „vor dem 1. Mai und 7. Oktober, da mussten wir auch immer eine Liste machen, wer so querulatorisch ist und immer große Reden führt, […] die mussten wir einladen, in die Beratungsstelle, und denen sagen: ‚Die sollen nicht mitmarschieren‘“ (041_F_20-12-10: 66). Diese Rolle der Fürsorger wurde von einer Kollegin aus Eberswalde bestätigt: „Ich kann mich erinnern, da trafen sich der Bundeskanzler Schmidt und Erich Honecker^3^. Dass wir alarmiert wurden, […] jetzt auffallende Menschen zu benennen“ (050_F_21-01-25: 76). Auch Begründungen für Ausgangsbeschränkungen oder Vorsorgeeinweisungen wurden genannt: „Weil die dann nicht irgendwo auffallen sollten“ (043_P_20-12-21: 48), erzählte eine ehemalige Pflegerin aus Eberswalde. Von Menschen, „die nicht ins Stadtbild passten“ (056_A_21-03-05: 36), sprach ein Arnsdorfer Arzt. „Die ganzen Heimbewohner und die Kranken […] das war für das […] Bild zum Demonstrieren nicht ganz gut“, erklärte eine Psychologin aus Ueckermünde (026_Psy_20-08-17: 133). Die „psychisch Kranken oder Behinderten“ sollten „in ihrem angestammten Raum bleiben und nicht raus dürfen“. Es sei „um das äußere Renommee“ gegangen, sagte ein Arzt aus Berlin (021_A_20-07-20: 91). Ein in Greifswald als Pfleger tätig Gewesener erinnerte sich, dass man nicht wollte, „dass da die […] psychisch Kranken und Behinderten mit rumzogen und der SED-Führung applaudierten“ (062_P_21-06-04: 55).

### Anweisende und Umgang mit den Anweisungen

Es gab von unterschiedlichen Stellen die Weisungen zur Aufnahme respektive Ausgangsbeschränkung zu speziellen Anlässen. Das zeigen die nachfolgenden Zitate. Ein Arzt aus Berlin äußerte sich: „die Polizei hat […] gesagt, also für diese Zeit soll der Patient doch möglichst nicht auffällig sein, sorgt dafür, dass der […] eingewiesen wird. Und da haben die Fürsorger dann oft […] versucht, so ein bisschen was Medizinisches reinzulegen, aber im Grunde war es eben klar, die Patienten sollten […] betreut werden, damit sie nicht draußen rumlaufen“ (021_A_20-07-20: 103). Andere Interviewte gaben an, dass die entsprechenden Anweisungen vom Vorgesetzten kamen, zum Beispiel dem Klinikdirektor, der sie vom Bezirksarzt erhalten habe. Eine Stralsunder Krankenschwester schilderte, dass die Anweisungen vom „Krankenhausleiter kamen und an den (Stations‑)Chef weitergegeben“ wurden (025_P_20-08-03: 203–220). Ein Arzt aus Berlin gab an, dass ihm der Klinikdirektor gesagt habe: „es gibt die Weisung vom Bezirksarzt, das kam von oben, […], das war die übliche staatliche Stelle“ (006_A_20-05-20: 37–38). Dann „gab es schon hin und wieder mal den Fall, dass wir von irgendeinem Kreisarzt einen Menschen eingewiesen bekamen“ (013_A_20-07-01: 209), sagte ein Greifswalder Arzt. Ebenso wurden das Ministerium für Staatssicherheit (MfS) und die Parteileitung der Sozialistischen Einheitspartei Deutschlands (SED) genannt. Ein Arzt aus Arnsdorf sagte: „Der [ärztliche Direktor] hat bekanntgegeben, dass es so erwünscht ist […] und der hat es bestimmt von der Staatssicherheit bekommen“ (032_A_20-10-12: 92–101).

Wie wurde mit solchen Anweisungen umgegangen und drohten Konsequenzen bei Zuwiderhandlung? Um das beantworten zu können, wurden die Aussagen der Berufsgruppen nach „Zuwiderhandlung“ beziehungsweise „Folgeleistung“ unterschieden (siehe Tab. 2).

**Tab. 2:** Nennung zum Umgang mit Anweisungen, getrennt nach Berufsgruppen (*n* = Anzahl der Sprecher)

Berufsgruppen	Umgang mit Anweisungen	Zuwiderhandlung	Folgeleistung
Ärzte	27 (*n* = 15)	22 (*n* = 11)	5 (*n* = 4)
Psychologen	6 (*n* = 4)	5 (*n* = 3)	1 (*n* = 1)
Fürsorger	4 (*n* = 3)	–	4 (*n* = 3)
Pflege	9 (*n* = 6)	–	9 (*n* = 6)
Zitate gesamt	46 (*n* = 28)	27 (*n* = 14)	19 (*n* = 14)

Ein Arzt aus Dresden berichtete, die Einweisungen zu diesen Veranstaltungen seien „systematisch gemacht worden“, aber man habe sich „erfolgreich gewehrt dagegen“. Man „konnte […] natürlich die Bettensituation vorschieben“ und sei „dann letztlich in Ruhe gelassen worden“ (058_A_21-03-24: 90). Ein Arzt aus Arnsdorf beschrieb: „Wer nicht psychisch krank ist, gehört nicht in ein psychiatrisches Krankenhaus“ (032_A20-10-12: 106–107), und ein Arzt aus Plauen äußerte: „Der Patient muss krank sein und es muss eine ernste Gefahr entweder für ihn oder für die Umgebung sein. […] Und dann bin ich auch nicht mehr belästigt worden“ (019_A_20-07-15: 155). Auch ein Brandenburger Arzt sagte: „da haben wir uns schon gehütet, nicht zum ausführenden Organ zu werden“ (015_A_20-07-06: 129). „Das hat keiner kontrolliert und auch keiner dann Rechenschaft abgeben müssen“, hieß es von einem Arzt aus Berlin (021_A_20-07-20: 103). Aber nicht alle beruflichen Akteure hatten diesen Handlungsspielraum, wie eine Ärztin aus Arnsdorf sagte: „In der Regel kamen die dann auch gleich mit einem Einweisungsgesetz versehen. Sodass wir nicht die Möglichkeit hatten zu sagen, der hat gar keine psychische Erkrankung, der geht jetzt wieder heim“ (056_A_21-03-05: 36). Von ähnlichen Erfahrungen wurde von einem Arzt aus Greifswald berichtet: „Und wie das so ist, wenn man einen Patienten kriegt, den muss ich erst mal angucken. Das dauert dann 1, 2, 3 Tage […] wir ahnten dann schon, worauf es hinausläuft. Aber erst einmal muss man den Patienten natürlich anschauen, bevor man da eine andere Diagnose trifft“ (013_A_20-07-01: 209). Eine Psychologin aus Ueckermünde berichtete von drohenden Konsequenzen: „Da gab es dann auch schon Ärger mit dem Bezirksarzt und der Partei“ (057_Psy_21-03-15: 32). An anderer Stelle wurde von einem Arzt aus Magdeburg angegeben, dass es „vorauseilender Gehorsam“ gewesen sei, ein „Vermeiden von Rüffeln“ (010_A_20-05-29: 223). Ein Psychologe, der in Rudolstadt tätig war, erinnerte sich, dass es „ein Kompromiss“ gewesen sei, denn „wenn wir das jetzt nicht machen, dann gibt es einen Haufen Trouble“ (036_Psy_20-11-16: 162). „Da konnte die Klinikleitung auch nichts gegen unternehmen, was sollten wir denn machen?“, schilderte eine Rostocker Fürsorgerin (002_F_2020-04-24: 102).

## Diskussion

**Abb. 1:**
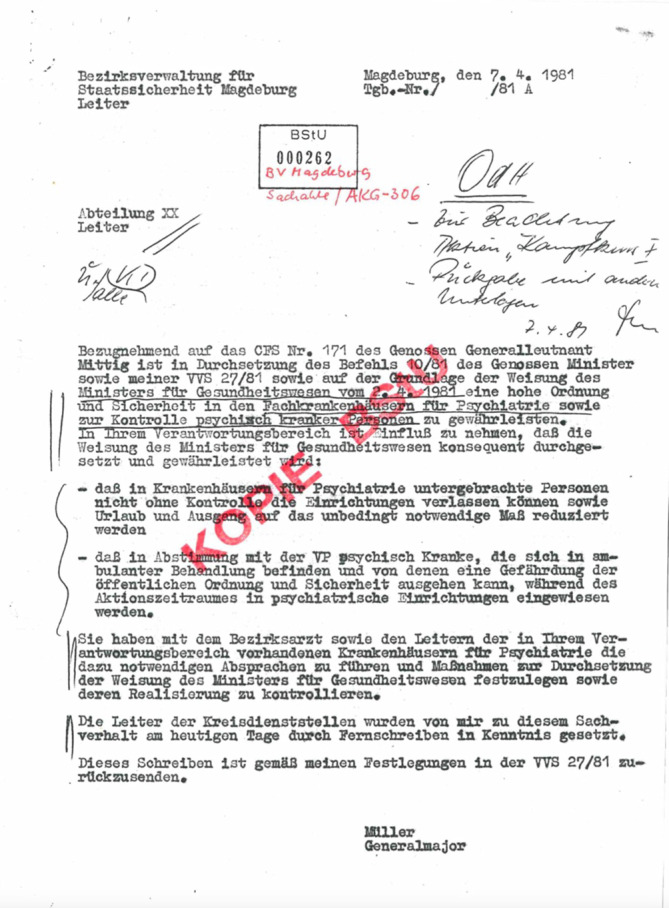
Anweisung der Staatssicherheit zur Durchsetzung der Nichtbeurlaubung bzw. Einweisung psychisch Kranker vom 07.04.1981 aus Anlass des X. Parteitags der SED (11.–16.04.1981), Quelle: BArch BStU, BV Magdeburg, AKG 306, Bl. 262

Aus den Berichten der Zeitzeugen wird ersichtlich, dass in der DDR psychisch kranke Menschen zu politisch motivierten Anlässen regelmäßig in psychiatrische Kliniken eingewiesen oder dort festgehalten werden sollten. Die Berichte sind eine gute Quelle, da sie andere Quellen, vor allem Archivalien, ergänzen [12]. Archivalische Hinweise, etwa in Form von Listen, finden sich nur sporadisch. So hat Süß auf ein Dokument der Erfassung von psychisch Kranken im Bestand der Staatsanwaltschaft in Berlin (Ost) aufmerksam gemacht [16]. Anlass waren die 1973 in Berlin stattgefundenen Weltfestspiele der Jugend und Studenten. Auch Erices konnte solche Hinweise in Archiven finden [3]. Sie scheinen aber eher unsystematisch zu sein, sind entweder schriftlich selten fixiert oder aber vernichtet worden. Eine von den Autoren gemachte Abfrage in den Kreisarchiven des ehemaligen Bezirks Schwerin brachte keine Anhaltspunkte. Lediglich ein Zufallsfund im Kreisarchiv Vorpommern-Greifswald gab einen Hinweis über eine Liste einzuweisender psychisch Kranker anlässlich des Besuchs von Helmut Schmidt in Güstrow 1981 [18]. Die erwähnte Liste war allerdings nicht auffindbar. Da schriftliche Überlieferungen zum großen Teil fehlen, erscheint es umso wichtiger, auf Zeitzeugen zurückzugreifen. Damit ist es besser möglich, die Anweisungen von der SED-Führung über die Ebenen der Staatssicherheit, Volkspolizei und der Bezirks- und Kreisärzte hin zu lokalen Entscheidungsträgern nachzuvollziehen (vgl. Abb. 1). Schließlich gab es Bestrebungen seitens der Staatssicherheit, alle psychisch kranken Menschen zentral erfassen und von „den behandelnden Ärzten spezielle Einschätzungen zu Personen, die zu Gewalttätigkeiten neigen“ [15Bl. 388], vornehmen zu lassen.

Die Interviews verdeutlichen, dass den Mitarbeitenden in den psychiatrischen Kliniken bewusst war, dass es Direktiven für medizinisch nicht indizierte Einweisungen und Beschränkungen gab. Der Umgang damit war unterschiedlich. Handlungsspielräume waren vorhanden. Diejenigen, die laut eigenen Angaben die Anweisungen ignorierten, berichteten von keinerlei schwerwiegenden Konsequenzen. Beachtet werden muss aber, dass die Kompetenzen für solche Entscheidungen im Allgemeinen der klinischen Hierarchie unterworfen waren.

### Einweisungsgesetz und Missbrauch der Psychiatrie in der DDR

Um die Erinnerungen der Zeitzeugen einordnen zu können, muss die Frage nach dem Verständnis von insbesondere politischem Missbrauch der Psychiatrie gestellt werden.

Das Vorgehen war nicht im Sinne des psychisch kranken Menschen, sondern im gesellschaftspolitischen Interesse. Ohne medizinische Indikation wurden sie anlässlich von gesellschaftlichen Ereignissen aus der Öffentlichkeit entfernt und in psychiatrische Kliniken eingewiesen oder dort zurückgehalten. Die Unterbringung und damit die Freiheitsentziehung wurde allein durch das Vorliegen einer psychischen Erkrankung gerechtfertigt. Deshalb muss von einem Missbrauch ausgegangen werden, der politisch motiviert war. Die nicht aus dem Krankheitsverlauf begründete Einweisung war eine Verletzung individueller Rechte. Laut Verfassung waren auch in der DDR die „Persönlichkeit und Freiheit jedes Bürgers […] unantastbar“ (Art. 30 [1]). Einschränkungen waren u. a. bei der Notwendigkeit einer „Heilbehandlung zulässig“ (Art. 30 [2]), die wiederum gesetzlich begründet werden musste. Geregelt wurde eine solche durch das 1968 in Kraft getretene „Gesetz über die Einweisung in stationäre Einrichtungen für psychisch Kranke“ [1]. Demnach konnten „psychisch Kranke, Kranke mit begründetem Verdacht auf eine psychische Erkrankung und Personen mit schwerer Fehlentwicklung der Persönlichkeit von Krankheitswert“ ohne deren Einwilligung oder die Einwilligung von gesetzlichen Vertretern eingewiesen werden, wenn der „Schutz […] des Kranken oder die Abwehr einer ernsten Gefahr für andere Personen oder für das Zusammenleben der Bürger“ bestand. Die Einweisung musste ärztlich angeordnet werden und über den Kreisarzt erfolgen. In der Klinik konnte der ärztliche Leiter mit Zustimmung des zuständigen Kreisarztes die Einweisung bzw. Unterbringung anordnen. Das Verfahren der Einweisung bzw. Unterbringung war also vor allem ein medizinisches. Eine Beschwerde konnte der Erkrankte oder der gesetzliche Vertreter innerhalb einer Woche einreichen [4]. Im Gegensatz zu den heutigen Psychisch-Kranken-Gesetzen der einzelnen Bundesländer war in der DDR ein richterlicher Beschluss erst nach sechs Wochen notwendig.

Die Zeitzeugenaussagen beziehen sich auf die 1970er- und 1980er-Jahre, deshalb kann von der Gültigkeit der genannten gesetzlichen Regelung ausgegangen werden. Ausschlaggebend ist, dass schon die Annahme eines bestimmten Verhaltens im Rahmen einer psychischen Erkrankung die Einweisung oder Beschränkung des Ausgangs begründen konnte. In den Interviews wird deutlich, dass es nicht um die Abwendung konkreter Gefahren für Patientinnen, Patienten oder andere Bürger ging, sondern primär darum, „Störungen“ der öffentlichen Ordnung zu vermeiden. Die Teilnahme psychisch erkrankter Menschen an öffentlichen Demonstrationen oder Feierlichkeiten konnte auch in der DDR nicht per se als „ernste Gefahr für das Zusammenleben der Bürger“ bewertet werden. Dies wurde ihnen aber unterstellt. Für die politischen Entscheidungsträger war ausschlaggebend, dass der Aufenthalt in einer psychiatrischen Klinik in zeitlicher Hinsicht ausreichte, um die Betroffenen von dem jeweiligen öffentlichen Ereignis fernzuhalten. Die Einweisung benötigte für die Prüfung in ärztlicher Hinsicht die Zeit, die zum Erreichen des politisch motivierten Ziels erforderlich war. Das stellt einen Missbrauch dar, in den die Mitarbeitenden in den Kliniken und insbesondere die Ärzte einbezogen wurden.

In Fachkreisen wurde aber immer wieder Kritik laut. Seit den 1970er-Jahren wurden vor allem von Psychiatern die Schwächen bei der Anwendung des Einweisungsgesetzes aufgezeigt [14]. Dazu gehörte u. a. die „großzügige Auslegung“ der „ernsten Gefahr für das Zusammenleben der Bürger“. 1984 war gefordert worden, die Häufigkeit der Zwangseinweisungen zu reduzieren und die Voraussetzungen bei jedem Eingewiesenen sorgfältig zu prüfen [8]. Einige verlangten, „die große Spielbreite der im gesellschaftlich zu tolerierenden Normbereich liegenden menschlichen Verhaltensweisen“ zu berücksichtigen, die eine psychiatrische Intervention nicht immer begründet [15]. Diese Forderungen können dahingehend aufgefasst werden, dass „ein Widerstand der Psychiatrie gegen einen erneuten Mißbrauch zum Zwecke der Disziplinierung […] mobilisiert werden sollte“ [1].

Nicht nur die Angst der SED-Eliten vor politisch unerwünschten Äußerungen und Taten psychisch kranker Menschen bildete ein Motiv des Wegsperrens in die Institution Psychiatrie. Staatsfeiertage wie der 1. Mai oder der 7. Oktober waren immer auch Stadtfeste mit Menschen und Begegnungen. Sie waren Höhepunkte im Krankenhausalltag. Die nicht tolerierte Anwesenheit von psychisch Kranken und Behinderten bei diesen staatspolitischen Anlässen entlarvt die Machthaber in der DDR als Vertreter einer „Diktatur des abstrakten Sollens – ‚So soll der Mensch sein!‘“ [9, S. 121].

### Die Verantwortung im Spannungsfeld zwischen Gesellschaft und Erkrankten

Was nicht beantwortet werden kann, ist die Frage, ob und inwieweit „Druck“ auf Patientinnen und Patienten ausgeübt wurde. Es bleibt unklar, ob etwa das Aufzeigen möglicher Konsequenzen oder die Vorgabe „medizinischer“ Gründe „freiwillig“ zu einer Einweisung oder dem Verbleib in der Klinik führte. Im Rahmen der therapeutischen Beziehung birgt eine paternalistische Haltung immer auch das Risiko der Bevormundung. Die Grenzen zum Missbrauch können verschwimmen. Um zu verstehen, warum Menschen in der DDR „funktioniert“ haben, kann es wichtig sein zu wissen, dass Konsequenzen drohen konnten, wenn Anweisungen nicht befolgt wurden. Auch wenn, wie sich die Zeitzeugen erinnerten, wenig oder nichts passierte, war das nicht klar und der Staat konnte folgenschwer in das Leben eines Menschen eingreifen. Das sollte andere warnen und führte zur Zurückhaltung, um sich selbst oder andere zu schützen. Die Anpassung an das autoritäre System begrenzte individuelle Möglichkeiten und war situationsabhängig, bot aber auch Spielraum für das eigene Handeln [5]. Das unterscheidet das Vorgehen von dem in der Sowjetunion, wo die Psychiatrie systematisch zur Verfolgung politischer Gegner missbraucht wurde.

## Fazit für die Praxis

Nach 35 Jahren hält die Diskussion über Anpassung und Widerstand in der DDR an. Mit der Psychiatrie findet sich ein besonders relevanter Bereich, um das Funktionieren und Wirken diktatorischer Strukturen besser zu verstehen. Der Blick in die psychiatrischen Institutionen und auf das Verhalten der Menschen innerhalb anderer gesellschaftlicher Rahmenbedingungen soll helfen zu verstehen, dass das Leben in der Diktatur moralisch nicht pauschal bewertet werden kann. Die Betrachtung jedes Einzelfalls und die Berücksichtigung der zu einer Entscheidung führenden Umstände sind wichtig. Auch heutzutage bedeutet das für die in der Psychiatrie Tätigen, im Wissen um die genannte Doppelfunktion wachsam zu sein, immer das Interesse des erkrankten Menschen zu beachten und gegenüber der Gesellschaft differenzierte Abwägungen zu vertreten. Schließlich gab und gibt es auch hierzulande Bestrebungen, psychisch kranke Menschen in einer zentralen Datei aufzulisten, um sie sicherheitspolitisch besser überwachen zu können. Dass das Vertrauen in den Staat mit einer solchen Maßnahme befördert wird, darf auch vor dem Hintergrund der DDR-Erfahrungen bezweifelt werden.

## Footnotes

1 Die Ergebnisse werden mit Zitaten aus den Zeitzeugenaussagen verdeutlicht. Nach den Zitaten ist die ID vermerkt. Ein „A“ steht für Ärztin/Arzt, „Psy“ für Psychologin/Psychologe, „P“ für Pflegende und „F“ für Fürsorgerin/Fürsorger. Nach dem Doppelpunkt steht die Nummer des Absatzes aus dem transkribierten Interview.

2 Damit sind die hohen Feiertage wie der 1. Mai („Internationaler Kampf- und Feiertag der Werktätigen für Frieden und Sozialismus“) und der 7. Oktober („Tag der Republik“) gemeint.

3 Bundeskanzler Helmut Schmidt besuchte am 13.12.1981 die DDR.
